# Rubella Seroprevalence and real-time PCR detection of RUBV among Congolese pregnant women

**DOI:** 10.1186/s12879-017-2352-6

**Published:** 2017-04-05

**Authors:** Josue Zanga, Makola Kennedy Mbanzulu, Arnold-Freddy Kabasele, Nlandu Roger Ngatu, Dimosi Roger Wumba

**Affiliations:** 1 Department of Tropical Medicine, Faculty of Medicine, University of Kinshasa, PoBox: 834, Kinshasa 11, Democratic Republic of the Congo; 2 Graduate School of Health Sciences & Nursing, University of Kochi, Ike campus, Kochi-city, Kochi prefecture Zipcode: 781-8515 Japan

**Keywords:** Democratic Republic of Congo, Kisantu town, Real-time PCR, Rubella virus infection, Seroprevalence

## Abstract

**Background:**

Rubella is an acute infectious disease caused by Rubella virus (RUBV). RUBV remains an important pathogen worldwide, causing approximately 100 000 cases of congenital rubella syndrome (CRS) every year; and the most severe consequence of rubella is teratogenicity. The aim of this study was to estimate the prevalence of RUBV IgG antibodies and determine RUBV genotypes in Congolese pregnant women in Kongo central province, Democratic Republic of Congo (DRC).

**Methods:**

This was a prospective cross-sectional study that consisted of a laboratory analysis of blood samples from 78 pregnant women to check for the presence of RUBV IgG antibodies, and also determine RUBV genotypes in seropositive samples (using primers targeting RUBV nucleoprotein), with the use of serological and molecular methods, respectively. Participants were pregnant women attending antenatal care clinics (ANC) at two health zones of Kisantu town in DRC. They were followed-up from the first to third trimester. Those who were negative for RUBV antibodies at the initial assay (first trimester) were tested in the second and, eventually, the third trimester.

**Results:**

An overall rubella seroprevalence of 58.97% was observed, whereas RUBV nucleoprotein was detected in 60% of randomly selected 30 blood samples among the 46 RUBV seropositive pregnant women. Five (27.77%) of positive samples were positive for both RUBV genotypes (RV8633/9112 and RV8945/9577), whereas 11 (61.11%) of them were positive for RV8633/9112 and two (11.11%) were positive for RV8945/9577 only. Regarding rubella clinical signs and complications, two subjects (2.56%) presented with fever, whereas five pregnant women (6.41%) had experienced abortion. None (0%) of the participants has been vaccinated against RUBV.

**Conclusions:**

Findings from this study suggest that RUBV is prevalent in Congolese pregnant women. Further research is required to elucidate the molecular epidemiology of RUBV in order to design a rational rubella surveillance and control program in DRC.

## Background

Rubella is an acute infectious disease that normally has a mild clinical course, caused by rubella virus (RUBV). RUBV remains an important pathogen worldwide, causing approximately 100 000 cases of congenital rubella syndrome every year [1, 2]. RUBV is classified into the family *Togaviridae.* The virus is roughly spherical with a diameter of 60-70 nm. *Togaviridae* family contains two genera, the Alphaviruses and the Rubivirus, RUBV being the sole member of the Rubivirus genus. The positive sense genome of RUBV consists of approximately 10,000 nucleotides and has one open reading frame (ORF) encoding the nonstructural proteins (NSPs) and one ORF for a subgenomic RNA encoding the structural proteins [3]. In the most recent WHO update, the standard nomenclature for the classification and designation of wild-type RUBV strains recognizes nine definitive and four provisional genotypes [4] expanding the nomenclature established in 2005 which was based on 739 nucleotides (nt) (nt 8731 to 9469) from the E1 gene sequence. This sequence encodes amino acids (aa) 159 to 404 (of the 481 aa) of the E1 glycoprotein. Although the knowledge of the geographic distribution of RUBV genotypes has grown substantially since 2003, the genotypes present in many countries and regions remain unknown [5], even though rubella is still recognized as a global public health issue [6].

The clinical manifestations of Rubella include a mild exanthema that is frequently accompanied by adenopathy and, occasionally, arthralgia. It can cause fetal death or congenital rubella syndrome (CRS) during early pregnancy (first trimester) which is characterized by multiple defects to the brain, heart, eyes and ears [1]. Encephalopathy and thrombocytopenia are rare complications of the disease. Itsmost severe consequence teratogenicity. This causes high neonatal morbidity and serious burden to families [7, 8].

Rubella has almost been eradicated by immunization programs in many developed countries, but outbreaks among unvaccinated individuals still occur [9]. The infection also continues to circulate in many countries with less effective immunization programs [10]. In some African countries, rubella seropositivity of 71-99% has been found in previous studies among women in their reproductive age, with countries like Mozambique (95%) andSouth Africa (97.5-98%) having highest incidence [11–13]. In 2011, a measles surveillance campaign implemented in the capital Kinshasa, Democratic Republic of Congo (DRC), showed that 24% of blood samples screened for measles were positive for rubella IgM antibodies [14].

Despite the severe consequences of rubella infection during early pregnancy, very little is known about the rubella seroprevalence in a number of African countries, DRC in particular. There is, therefore, a continued demand for the assessment of pregnant women who develop or have contact with rubella-like illnesses. The diagnosis of rubella is currently made by using serological techniques, and the risk to the fetus is assessed by establishing the gestational age at the time of maternal infection. Considering the negative health consequences of RUBV on the fetus and offspring of infected mothers, an early laboratory diagnosis of recent or congenital infection through direct detection of RUBV ribonucleic acid (RNA) in clinical specimens, in addition to the detection of RUBV-specific immunoglobin M (IgM), is obviously critical [15].

The World Health Organization (WHO) aims to eliminate measles and rubella and to reduce the incidence of CRS to less than one case per 100,000 live births [16]. For this purpose, epidemiological surveillance based on the laboratory diagnosis and the characterization of the genotype of circulating strains are included in the WHO’s recommendations. Between 2001 and 2008, a WHO initiative against rubella has provided support to developing countries including the DRC. However, this initiative experienced non-continuity due to lower investment and the lack of political resolve that has led to inadequate vaccination coverage in DRC for a number of years.

To our knowledge, there have been no studies that prospectively investigated rubella seroprevalence and performed the molecular detection of circulating RUBV strains among Congolese women attending antenatal care (ANC) clinics. The aim of the present study was to prospectively estimate rubella seroprevalence and determine RUBV genotypes among Congolese pregnant women in Kisantu town, Kongo central province, DRC.

## Methods

### Study design, sites and participants

This was a prospective cross-sectional study that consisted of a laboratory analysis of blood samples from pregnant women undergoing antenatal care (ANC) (N1 = 78) to check for the presence of rubella virus IgG antibodies and also RUBV genotypes in RUBV seropositive subjects, with the use of serological and molecular methods, respectively. DRC is a country located in the African Great Lakes region. It is the second largest country in Africa with an estimated population of over 75 million [17]. This study was conducted in the Congolese town of Kisantu, Kongo central province (formerly Bas-Congo), from 1 February through March 2014.

**Fig. 1:**
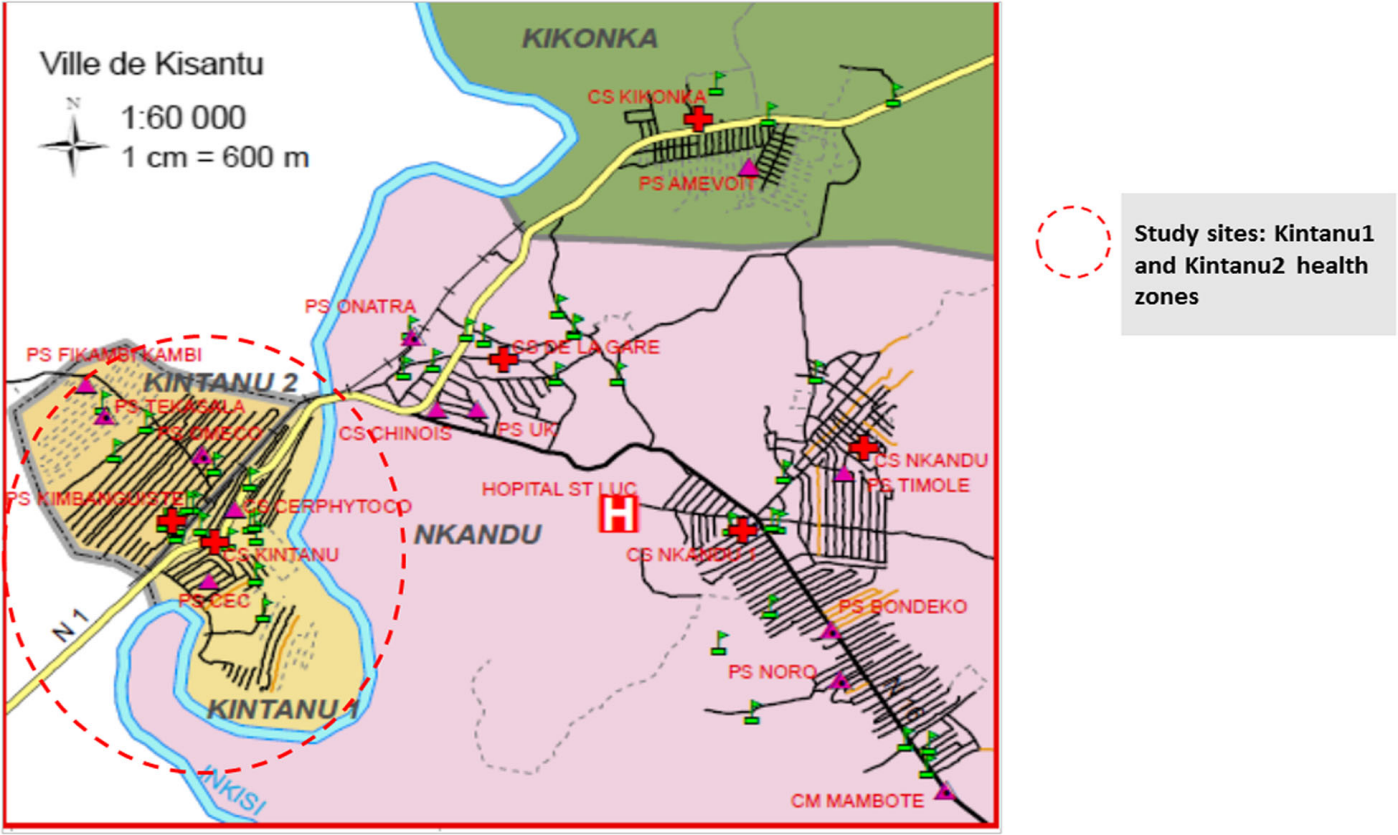
Map of Kisantu showing health settings within Kisantu town and the 2 study sites, Kintanu1 and Kintanu2 *(Courtesy of Central Bureau of Kisantu health zone, Kongo central, Democratic Republic of Congo*)

Kisantu is located approximately at 110-115 Km from the capital Kinshasa. According to the current Congolese health system, Kisantu is divided into four ‘health zones’, including Nkandu, Kikonka, Kintanu 1 and Kintanu 2. Each health zones comprises one or more health centers. The present study was conducted at the antenatal care clinics (ANC) of Kintanu 1 and 2 health centers (Fig. 1). A consecutive sampling technique was applied in the population of pregnant women visiting ANC clinics in selected study sites. Participants were interviewed and the information obtained was recorded in an epidemiological form. They also provided blood samples for serological analysis, and a randomly selected sample of blood specimens from RUBV seropositive pregnant women was used to determine RUBV genotypes.

### Procedures

#### Detection of IgG anti-rubella

Blood samples were collected from pregnant woman attending ANC in Kisantu. After collection, blood samples were transported on ice in cold boxes to the Saint Luc laboratory in Kisantu for the serodiagnosis of rubella virus infection. Blood samples were allowed to clot and centrifuged for serum separation prior to testing. All sera were stored at −20 °C until use. Evidence of presence rubella contamination was tested using an IgG enzyme-linked immunoassay to detect rubella-specific IgG antibodies, according to the manufacturer’s specifications (Wampole Laboratories, Cranbury, New Jersey, United States of America USA). The limit of detection of the test was 6.2 IU/mL. Individuals with an IgG titer >8.6 IU/mL were defined as seropositive, whereas titers between 6.2–8.6 IU/mL were classified as borderline.

#### Molecular analysis

Before clotting, blood samples were spotted onto FTA cards for molecular analysis. FTA cards were allowed to dry and stored at room temperature before they were transported to SUA for RUBV detection using RT-PCR.

- *RNA extraction*

A sterile scapel blade was used to remove 25 mm discs from the center of each dried sample spot on the FTA Card. Viral RNA was recovered from sera using QIAamp Viral RNA Mini extraction kit (Qiagen, Hilden, Germany), according to the manufacturer’s instructions (Appendix 2). Briefly, samples were lysed using a lysis buffer to lyse cells and viral envelope followed with chemical protein precipitation using ethanol. Protein precipitates were pelleted by centrifugationand the supernatant was passed through a silica column to trap the RNA.Column-bound RNA were washed with two different buffers and the column dried by high speed centrifugation. Afterwards, column-bound RNA was eluted with RNase free water. Extracted viral RNA was stored at −80 °C until RT-PCR.

b) *Reverse transcription polymerase chain reaction (RT-PCR) for the detection of RUBV*

RT-PCR for the detection of RUBV was carried out in a GeneAmp PCR system 9700 (Applied Biosystems, Carlsbad, USA) using AgPath-ID one-step RT-PCR kit (Applied Biosystems, Courtaboeuf, France). The RT-PCR master mix for a single reaction is shown in Table 1. Briefly, reverse transcription was performed using a reverse transcriptase for 30 min at 50 °C followed by initial denaturation of DNA for 15 min at 95 °C. Afterwards, 40 PCR cycles consisting of denaturation of DNA at 94 °C for 30 s, annealing of primers at 55 °C for 30 s and DNA extension by a DNA polymerase for 60 s. PCR was followed by a final extension at 72 °C for 10 min. Partial RUBV amplification was performed using either RUBV primer pair RV 8633 and RV 8945 or RV 9112 and RV 9577.

**Table 1:** Reverse transcription polymerase chain reaction (RT-PCR) master mix components for a single reaction carried out using AgPath-ID one–step RT-PCR kit from Applied Biosystems to detect RUBV in FTA cards

No	Component	Volume (mL)
1	2 X RT-PCR Buffer	12▪5
2	10 μM RV8633 or RV9112	1
3	10 μM RV8945 or RV9577	1
4	Nuclease-free water	9
5	25XRT-PCR Enzyme Mix	0.5
6	Extracted DNA template	1
	Total volume per reaction	25▪0

RT-PCR products were separated by electrophoresis on a 1·5% agarose gel in 0·5%TBE buffer (SERVA Electrophoresis, Heidelberg, Germany) stained with GelRed nucleic acid stain (Phenix Research Products, Candler, USA). Each well was loaded with 5 μl of the PCR product and 3 μl of blue / orange 6X DNA loading dye (Promega, Madison, USA). Samples were separated along with a 1000 bp DNA ladder (Promega, Madison, USA) at 150 V for 30 min. The agarose gel was visualized using a gel documentation system (EZ gel doc BioRad, France).

### Ethical consideration and data analysis

The study protocol was approved by the ethics committee of Sokoine University, Tanzania, and a written authorization was obtained from the Kisantu area health authority. Informed consent was obtained from each participant; those who did not consent were excluded from the study, but none of them was penalized. Qualitative data are presented as proportions. Fisher’s exact test was performed to assess the relationship between any two categorical variables, whereas logistic regression test was used to assess the correlation between rubella seropositivity and participants’ characteristics. The analyses were performed using Epi info version 7 software. For the different statistic tests (bivariate analysis), the level of significance was set at 5% (2- tailed p).

## Results

### Socio-demographic characteristics of participants (Table 2)

In the present study, a total of seventy eight pregnant women were enrolled after obtaining their consent. The enrolled pregnant women were aged 14-43 years with a median age of 25 years; 30.77% (24/78) of women were primigravidea. The majority (75.64%; 59/78) of the enrolled subjects were married, whereas only 24.36% (19/78) subjects were single. The education level differed among subjects including university education (6.41%; 5/78) and high school education (78.21%; 61/78). Most pregnant women enrolled in this study were from Kintanu 2; the group of those who were in their first trimester of gestational age comprised 11 subjects (14.10%). None of the pregnant women enrolled in this study has been vaccinated against rubella.

**Table 2:** Socio-demographic and obstetrical characteristics of participants

Sociodemographics		*n* (%)
Age	< 30 y.	58 (74▪36)
	30 - 43 y.	20 (25▪64)
Parity	Primigravid	24 (30▪77)
	Multigravid	54 (69▪23)
Gest. age	1st Trimester	11 (14▪10)
	2nd & 3rd Trimester	67 (85▪90)
Marital status	Single	19 (24▪36)
	Married	59 (75▪64)
Occupation	Presence of activity	47 (60▪26)
	Housewife	31 (39▪05)
Residence	Kintanu 1	25 (32▪05)
	Kintanu 2	53 (67▪95)

### Rubella clinical signs and seroprevalence of RUBV antibodies

Table 3 shows the relationship between rubella seropositivity and the sociodemographic and obstetrical characteristics of the study participants, namely age, marital status, occupation, parity/gravidity and gestational age. No association was found between the serological status of the pregnant women with either of the participants’ characteristics (Table 3).

**Table 3:** Correlation between rubella and characteristics of study participants

Sociodemographic and obstetrical characteristics		Rubella IgG positive	OR	95% Cl	*P*-value
		*n* (%)			
Age	<30	36	1▪23	0▪34 - 4▪45	0▪99
	30-43	10	-	-	
Parity/gravidity	Primigravid	16	-	-	
	Multigravid	30	0▪62	0▪22 - 1▪70	0▪51
Gestational age	Trimester 1	6	-	-	
	Trimester 2&3	40	1▪23	0▪34 - 4▪45	0▪99
Marital status	Single	13	-	-	
	Married	33	0▪58	0▪19 - 1▪70	0▪48
Occupation	Presence of job	26	0▪59	0▪22 - 1▪52	0▪39
	Housewife	20	-	-	

**Fig. 2:**
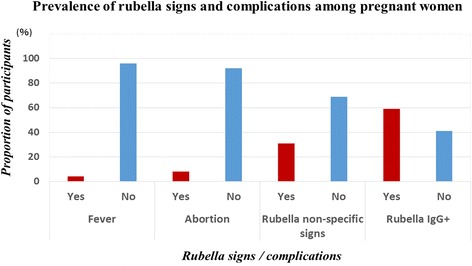
Clinical signs, complications and rubella seroprevalence among participants

Regarding rubella clinical signs or complications in the study participants, fever was observed in two subjects (2.56%), whereas five pregnant women (6.41%) had experienced abortion. Non-specific rubella signs were found in 10.26% (8/78) of participants. On the other hand, of the 78 serum samples from the study participants that were tested for the presence of RUBV antibodies, the seroprevalence of rubella IgG antibodies was 58.97% (46/78) (Fig. 2).

### Confirmation of RUBV by RT-PCR

**Fig. 3:**
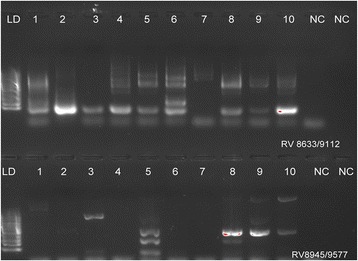
Rubella virus (RUBV) detection in blood samples from pregnant women using RT-PCR

A total of 30 samples were randomly selected and tested for RUBV using RT-PCR. RUBV was detected in 18 out of 30 samples (60%) (Fig. 3). Of the 18 positive samples, eight (44.44%) were from Kintanu 1 and ten (55.55%) from Kintanu 2. Regarding RUBV genotypes, of the 18 positive samples, 11 (61.11%) were positive when the primer pair RV 8633/9112 was used, whereas two (11.11%) were positive using the RV 8945/9577 primer pair. In addition, five samples (27.77%) were positive on both RV 8633/9112 and RV8945/9577 primer pairs.

## Discussion

This study determined the seroprevalence of rubella among Congolese pregnant women attending ANC in Kisantu town, DRC, with the use of serological and molecular methods. Antibodies (IgG) against RUBVwere detected using ELISA, whereas RUBV genome was detected using RT-PCR. The findings showed that more than half (58.97%) of pregnant women were seropositive for rubella virus. This seroprevalence is similar to results from two other studies carried out in Sudan and Nigeria, which showed a seroprevalence of 65-68%, respectively [18–21]. On the other hand, studies conducted in Burkinafaso, Senegal and Iran showed higher rubella seroprevalence (95.0, 90.1 and 96.2%, respectively) among pregnant women [22, 23]. Another study conducted among urban and rural pregnant women in Namibia showed an overall rubella seroprevalence of 85%, with urban women having higher risk of infection [24]. These differences in seroprevalence rates can be attributed to the degree of contamination at national level, the testing method used and/or the size of the sample used.

Since rubella vaccine is rarely given to adults in DRC, our results suggest that a sizable number of women become infected during child-bearing age. The mostexposed age group being between 14 to 29 years of age. According to a study conducted in Cameroon in 2011, the age group of women from 20 to 39 was considered to have a maximum fertility [25]. Our findings highlight the need for the country to establish surveillance of trends in susceptibility to rubella and CRS incidence and introduce of rubella vaccination among women of childbearing age.

Our study also showed that neither age, gestational age, gravidity, marital status nor occupation was significantly associated with presence of RUBV IgG. Studies conducted in Burkinafaso and Sudan also showed no correlations between rates of rubella seropositivity and educational, marital, and pregnancy status, monthly income or history of previous exanthematous diseases [18, 23]. This lack ofrelationship seems toaffectall studiesdespitethe size of thesample, the location ofthe studyorthevariationof gestational age. Nevertheless, the overall results indicate that a considerable proportion of pregnant women in DRC are at risk of primary infection with rubella virus.

Widespread use of rubella vaccine in most developed countries has contributed to markedly reducing rubella incidence. Since the 1970’s, in many of those countries, vaccination programmes have been targeting pre-adolescent girls before they become sexually active [19]. In contrast, in several countries of the developing world, rubella immunization programme does not exist. Of the 46 African countries covered by the WHO measles prevention programme, only two countries, namely Mauritius and Seychelles, have included routine rubella vaccine in their national vaccination programmes [13, 20]. Our study showed that none of the participating pregnant women has been immunized against rubella. This confirms that rubella vaccination is not part of activities included in ANC clinics in DRC, and particularly in Kongo central province where the study was conducted.

### Limitations and strengths

This study has some limitations. The sample was relatively low, 78 pregnant women. This was a study in which participants were enrolled prospectively, and according to our study protocol, we expected a least 100 pregnant women undergoing ANC would be enrolled within the 2-month period of the study, given the relatively high natality rate in DRC. However, a number of factors might have affected pregnant women’s willingness to participate in this study. Firstly, participation was voluntary and only those who freely accepted to take part and accepted to undergo blood sampling and testing were eligible. It is well known that in rural Congo, as well in most other areas in the Sub-Saharan African countries, medical procedures such as vaccination and blood sampling for reasons other than hospital-based patient’s care face opposition from community members’ beliefs. Moreover, the sample size may have also been affected by the accessibility to ANC in the context of poverty in rural Kongo central (Bakongo) where this study was carried out. In fact, ANC accessibility is limited as, depending on household socioeconomic status, the choice between health center and rural or village birth attendant is decided.

Another fact is that, of the 78 blood samples, 30 randomly selected samples were assayed to determine the RUBV genotypes in study participants. RT-PCR was a complementary assay performed not systematically for all 78 participants but only for those who were positive in the serological analysis (RUBV antibodies); thus, the randomization concerned not 78 samples but 46 ‘RUBV antibody’ positive samples, of which 30 were randomly drawn for RT-PCR with the purpose of identifying RUBV genotypes.

Nonetheless, this prospective study provides new knowledge in regard to the trend of rubella seroprevalence among Congolese pregnant women and RUBV genotypes circulating in the region. Thus, it may serve as a reference for future research projects on rubella and, eventually, the design of rubella surveillance and control program of in DRC.

## Conclusions

As a conclusion, the present study showedthat RUBV is circulating widely in pregnant women in DRC and that RUBV is present in Kisantu area in Bas Congo region. The antibodies and genome of RUBV were detected by ELISA (IgG) and RT-PCR. The results of the study also confirm for the first time the presence of RUBV genome in Kisantu, DRC. Based on these findings, it should be recommended that serological and molecular screening tools should be used in surveillance for RUBV in DRC, especially in pregnant women in order to control rubella. A high seropositivity in the absence of vaccination indicates a high risk posed to newborns due to CRS. More studies on the epidemiologyof RUBV are recommended to gather more data for effective control measures including characterizing the strains that can be used for development of specific vaccine.

## Abbreviations

ANC: Antenatal care
CRS: Congenital rubella syndrome
DRC: Democratic Republic of Congo
ELISA: Enzyme linked immunosorbent assay
RT-PCR: Reverse transcription polymerase chain reaction
RUBV: Rubella virus
